# Self-Nanoemulsifying System Loaded with Sildenafil Citrate and Incorporated within Oral Lyophilized Flash Tablets: Preparation, Optimization, and In Vivo Evaluation

**DOI:** 10.3390/pharmaceutics12111124

**Published:** 2020-11-21

**Authors:** Khaled M. Hosny, Nabil A. Alhakamy, Maeen A. Almodhwahi, Mallesh Kurakula, Alshaimaa M. Almehmady, Samar S. Elgebaly

**Affiliations:** 1 Department of Pharmaceutics, Faculty of Pharmacy, King Abdulaziz University, Jeddah 21589, Saudi Arabia; nalhakamy@kau.edu.sa (N.A.A.); ph.maeenam@gmail.com (M.A.A.); amnalmehmady@kau.edu.sa (A.M.A.); 2 Center of Excellence for Drug Research and Pharmaceutical Industries, King Abdulaziz University, Jeddah 21589, Saudi Arabia; 3 Department of Pharmaceutics and Industrial Pharmacy, Faculty of Pharmacy, Beni Suef University, Beni Suef 62511, Egypt; 4 Department of Biomedical Engineering, The Herff College of Engineering, University of Memphis, Memphis, TN 38152, USA; m.kurakula@memphis.edu; 5 Department of Medical Engineer, Elko-Medical Company, Algomhoria Street, Cairo 22132, Egypt; beautsway@yahoo.com

**Keywords:** bioavailability, sildenafil citrate, erectile dysfunction, lyophilized tablets, self-nanoemulsifying lyophilized tablet

## Abstract

Sildenafil citrate is a drug used throughout the world primarily to treat erectile dysfunction. Several problems with the commercially available product decrease its efficacy, such as limited solubility, delayed onset of action, and low bioavailability with a large variability in the absorption profile. This study aimed to develop an optimized self-nanoemulsifying lyophilized tablet for the drug to conquer the foresaid problems. Sildenafil solubility in various surfactants, oils, and cosurfactants was attempted. An optimized formulation of a loaded self-nanoemulsion with a small droplet size was developed by applying a special cubic model of the mixture design. Sixteen formulations were prepared and characterized for droplet size. On the basis of solubility studies, a clove oil/oleic acid mixture, polysorbate 20 (Tween 20), and propylene glycol were selected as the proposed oil, surfactant, and cosurfactant, respectively. On the basis of desirability, an optimized sildenafil citrate-loaded self-nanoemulsifying delivery system containing 10% of the oil mixture, 60% of the surfactant, and 30% of the cosurfactant had a droplet size of 65 nm. Subsequently, the tablet form was fabricated with optimum ratios of 0.4% fumed silica, 0.1% hydroxypropyl methylcellulose, and 0.4% sodium starch glycolate. This formula showed satisfactory results in both disintegration and dissolution studies. In vivo pharmacokinetic studies indicated a higher bioavailability (1.44 times) and rapid absorption profile for the study’s tablets compared with commercially available tablets. In conclusion, highly bioavailable oral lyophilized flash tablets of sildenafil were successfully prepared. They will be a good alternative to the conventional solid-dosage form.

## 1. Introduction

The lack of ability of men to accomplish or sustain an erection that will complete sexual activity is called erectile dysfunction (ED). ED has affected roughly 20 to 30 million men in the United States alone [1]. It is a complex disorder resulting from both physical and psychological disorders. Physical causes include neurologic, muscular, circulatory, nervous system, and endocrinological disorders, and psychological causes occur because of emotional disorders. Medication side effects may also be a cause [2]. Psychogenic reasons are the main reasons for this complicated situation. Stimulatory and inhibitory functions are impaired, and this affects penile erection negatively. Ten to nineteen percent of cases of ED have a neurogenic origin, as erection is a neurovascular event [3]. Physiologic issues that may lead to ED include heart disease, atherosclerosis, tobacco use, obesity, low testosterone, and injuries that affect the pelvic area or spinal cord [4].

Sildenafil citrate (SLC) is a salt of a pyrazolopyrimidine derivative having a structure similar to zaprinast and used to treat ED. It has a structure similar to the cyclic guanosine monophosphate that is responsible for vasodilation effects [5]. SLC is also used to treat patients who undergo bilateral nerve-sparing radical retropubic prostatectomy. Loss of erection may occur as a side effect after prostatectomy surgery and the recovery process of the normal action is slow [6]. The ability of normal erection to return is enhanced when a patient is treated proactively and administered SLC at bedtime [7].

ED affects diabetic patients owing to several complex mechanisms such as vascular diseases, neuropathy, psychopathy, or hormone fluctuations. SLC improves ED in diabetes mellitus type II, which occurs independently of the glycemic level and vascular complications [8]. Plasma concentrations of SLC reach the maximum level 1 to 1.5 h after oral ingestion and have a half-life of 3 to 6 h. SLC is cleared from the body mainly via the liver, and it has 40% oral bioavailability [9]. Bioavailability of the SLC tablet may be decreased due to many reasons, such as elevation of hepatic transaminase, aging, and taking concomitant drugs [10]. Food also affects the bioavailability of SLC [11]; it is reported that the presence of food delays the absorption of SLC, and this minimizes both the T*_max_* and C*_max_* of SLC. The first-pass effect also reduces SLC bioavailability [12]; studies observed that 49% of oral SLC tablets are affected by the hepatic and intestinal first-pass effect.

Nanotechnology is one of the promising technologies in the twenty-first century that has potential applications, especially in the area of formulation development. Nanotechnology has emerged as a state-of-the-art solution for the majority of diagnostic and therapeutic agents and is also capable of improving the efficacy and safety of many drugs [13,14]. The self-nanoemulsifying delivery system (SNED), on dilution, produces nanosized transparent systems known as nanoemulsions (NEs). These systems were studied extensively because of their potential applications in the design of dosage forms [15,16,17,18,19,20]. The self-nanoemulsifying drug delivery system SNEDDS can offer higher stability (in contrast with other lipid-based systems) [21,22] and improved solubility, and thus they augment the oral bioavailability of many lipophilic drugs [23]. In general, SNEDDS can be formed as capsules for ease of administration. Yet, this may have a few issues such as leakage of liquid from the capsule, incompatibility with the capsule shell, and an expensive capsule liquid filling technology [24]. To overcome these drawbacks, solid carriers were utilized to solidify the SNEDDS; this could further enhance patient convenience, dose accuracy, and stability [25,26,27]. Several porous carriers such as calcium silicate, magnesium aluminometasilicate, silicon dioxide, and dibasic calcium phosphate were used effectively for the solidification of SNEDDS, because they were capable of adsorbing the liquid [28,29,30,31,32]. Lyophilization methodology offers tablets with a porous structure, which leads to quick penetration of saliva when placed in the oral cavity. Moreover, lyophilization prolongs the shelf-life of the product during storage. Cryoprotectants such as sugars were used for dehydration of the prepared tablets [33].

There are a few challenges associated with the oral administration of SLC, including poor bioavailability and short duration of action that requires frequent administration. The aim of the current study was to enhance the bioavailability and solubility of SLC by employing a mixture design through improving their oral disintegration and dissolution. The current study started with enhancing the bioavailability through the development of an optimized SLC-loaded SNED as a first step. During this step, SLC solubility in different surfactants, oils, and cosurfactants was estimated. A special cubic model of the mixture design was applied to develop an optimized SLC-loaded SNED with a minimum droplet size. Sixteen formulations were prepared and characterized for droplet size. The second step was to prepare and optimize the self-nanoemulsifying lyophilized tablets (SNTs) loaded with the SLC SNED using the freeze-drying technique. Tablet formulations used additives such as fumed silica, hydroxypropyl methylcellulose (HPMC), and sodium starch glycolate (Explotab^®^). Each tablet was evaluated to determine the disintegration time and percentage released after 5 min.

The final stage was to evaluate the in vivo pharmacokinetic parameters (C*_max_*, T*_max_*, and area under the curve (AUC)) of the optimized SNTs and compare the results with the pharmacokinetic findings of commercially available tablets, to monitor the bioavailability improvement.

## 2. Materials and Methods

### 2.1. Materials

SLC was procured from Xian Sunny Biochemical Technology (Shaanxi, China). The clove oil, propylene glycol, and Explotabs were procured from the Tedia Company (Fairfield, OH, USA) and JRS Pharma (Patterson, NY, USA). Different grades of polyethylene glycol (PEG-200, PEG-400), Labrasol, Transcutol HP, Lauroglycol 90, Maisine cc, Capryol 90, Labrafac PG, and Labrafac lipophile WL1349 were generously gifted by Gattefosse (Saint-Priest, France). Tween 20, Triacetin, sesame oil, oleic acid, olive oil, fumed silica (0.007 µm), microcrystalline cellulose (Avicel^®^ PH-101), gelatin, mannitol, castor oil, methanol, and HPMC were purchased from Sigma-Aldrich (St. Louis, MO, USA). All additional reagents and chemicals used were of standard analytical grades.

### 2.2. Estimation of Sildenafil Citrate (SLC) Solubility in Various SNEDDS Components

The SLC solubility was determined in assorted oils, such as oleic acid, castor oil, clove oil, olive oil, Triacetin, Capryol 90, Maisine cc, and sesame oil and also in various surfactants such as Tween 20, Labrafac PG, Labrafac lipophile WL-1349, Lauroglycol 90, and Labrasol. SLC solubility was studied in various cosurfactants such as propylene glycol, PEG-200 and PEG-400, and Transcutol HP. An excess amount of SLC was dissolved in 2 mL of all the selected liquid systems, and the formed blend was mixed thoroughly by keeping it on a water bath (Model 1031, GFL Corporation, Burgwedel, Germany) at 25 ± 0.5 °C for 48 h. When the mixture reached equilibrium, approximately 1 mL of the sample was collected and centrifuged (AIC Micro Centrifuge- Boerne, TX USA) for approximately 15 min at 1000 rpm. Supernatants were collected and diluted with methanol as required. A concentration of SLC was quantified in the final sample by spectrophotometric analysis (Jenway 7315; Bibby Scientific Limited, Stone, UK) at 239 nm. All the experiments were carried out in triplicate [34].

### 2.3. Pseudoternary-Phase Diagram for SLC in Various Solvent Systems

A ternary-phase diagram of SLC was constructed on the basis of SLC solubility in the selected systems (a mixture of oils, clove oil/oleic acid, surfactant, Tween 20, and cosurfactant, propylene glycol) to identify the suitable levels for formulating a self-nanoemulsion component. The dilution test with water is the preliminary test for mixture design, and it should be capable of forming a clear NE on dilution. The total concentration of the three parameters was maintained at 100%. Various combinations were identified to study the NE region in the generated pseudoternary-phase diagram.

### 2.4. Optimization of Preparation of SLC-Loaded SNEDDS as per Mixture Design

The experimental mixture design has proven to be an efficient tool owing to its precise methodology, and, hence, it was applied for the optimization of the SNEDDS preparation. Among several models, a special cubic model of extreme vertices was selected in order to determine the main and interaction effects of proposed variables on the selected responses. The three independent variables were the concentrations of the oil mixture (clove oil/oleic acid) (X_1_), Tween 20 (X_2_), and propylene glycol (X_3_). All these factors were used in different ratios and the total concentration was maintained at 100%. The mean globule size was selected as the dependent variable/response (Y). A total of 16 runs were projected with a randomized order (50 mg of SLC in each formulation). The summary of variables with their levels selected for NE formulation is shown in Table 1. About 1 g of each mixture was prepared by simple mixing of selected components in a 2-mL Eppendorf tube by vortex mixture for a minute. The total of three components in the mixture will be always equal to 100%. The formulation composition of SNEDDS and the mean of the observed globular size are shown in Table 2. The relationship between the selected factors and the evaluated responses was further studied with regression equation and statistical analysis strategies by applying Statgraphics Centurion XV software (v. 15.2.05, StatPoint Technologies, Warrenton, VA, USA). All the formulated dispersions were estimated for the ability to emulsify and the appearance of formed NE. The formulation with the smallest globule size was selected as the optimum formulation and consequently used for the preparation of the self-nanoemulsifying lyophilized tablets (SLC-SNTs).

### 2.5. Assessment of the SLC-SNEDDS

#### 2.5.1. Emulsification Ability

The efficiency of the prepared SLC-SNED was assessed visually for its instant emulsification ability and clarity of the formed emulsion.

#### 2.5.2. Determination of Globule Size of the Emulsion

About 125 mg of the formulated dispersion was diluted with the required amount of distilled water to form an aliquot of 20 mL, and the prepared sample was utilized to analyze the globule size by the dynamic light-scattering technique (Zetatrac, Microtrac, Montgomeryville, PA, USA).

#### 2.5.3. Stability Studies

The formulation of SLC-SNEDDS was subjected to various temperature changes to confirm its thermodynamic stability. SNEDDS formulations were exposed to three consecutive freeze–thaw cycles (freezing at −25 °C for approximately 12 h and thawing at +25 °C for 12 h). After consecutive cycles, the final product was analyzed for globule size. The stability index was calculated by comparing the globule size with that in initial reports, using the following equation:

Stability index = ([Initial size − Change in size]/Initial size) × 100(1)

#### 2.5.4. Surface Morphology of SLC-SNEDDS

Structural and morphologic observations of globules were studied using a transmission electron microscope (H7500, Hitachi, Japan). Initially, the sample was diluted with distilled water approximately 200 times and then positioned on the microscopic grids. Additionally, a 0.5% phosphotungstic acid solution was used to stain the sample and then dried effectively. The dried sample was analyzed by operating the TEM at 80 kV with darkfield imaging with increasing magnification.

### 2.6. Preparation of SLC-SNTs

SLC-SNTs were formulated and prepared as per the method portrayed earlier by El-say KM et al. Each formulation contained 50 mg of SLC, added to 1 g of the optimized SLC-SNED, and then the gelatin solution was mixed (2% 9 mL) on a stirrer in addition to a constant amount of 400 mg of fumed silica, 100 mg of HPMC, 400 mg of Explotab, 100 mg of diluent (Avicel^®^ PH 101), and 100 mg of mannitol. Initially, the formulation was mixed for 2 min by Vortex and subsequently by a probe sonicator (Sonics Vibra-Cell VCX 750, Sonics and Materials, Inc., Newtown, CT, USA) until a homogenous mixture was produced. The prepared mixture was placed into tablet blister pockets and transferred to and stored in the freezer for 24 h at −22 °C. Afterward, the sample was freeze-dried in Christ Alpha 1-2 LD Plus lyophilizer (Osterode, Germany). Parameters maintained for freeze-drying were (1) a time of 24 h, (2) a condenser temperature of −45 °C, and (3) a pressure of 7 × 10^−2^ mbar. The final product was in the form of a patch. The patch was made into a tablet by adding suitable concentrations of diluents and disintegrating agents. The final lyophilized tablets were evaluated for disintegration and dissolution profiles.

### 2.7. Optimization of SLC-SNTs

The results obtained from the 15 formulations were further statistically analyzed by the analysis of variance (ANOVA). The formulation was optimized with three variables to acquire less disintegration time with the desired drug release profile. Accordingly, the optimized formulation was evaluated for disintegration and dissolution to compare the predicted and experimental values.

### 2.8. Wetting Time

Wetting time of SLC-SNTs was estimated by placing the piece of twice folded tissue paper (12 cm × 10.75 cm) in a small Petri dish of internal diameter 6.5 cm. Subsequently, 6 mL of Sorenson’s buffer solution of pH 6.8 was added. A formulated tablet was placed in the paper and the time taken for complete wetting was determined (Mean ± S.D, *n* = 3).

### 2.9. Disintegration Time

Disintegration time of the prepared SLC-SNTs was determined using PharmaTest disintegration tester (Hainburg, Germany) with six tablets; 900 mL of distilled water at 37 ± 0.5 °C was used as media as per USP specifications. The disintegration time was considered as the time passed until there were no residue or only a trace amount of soft residue on the screen (#10) (Mean ± S.D, *n* = 6).

### 2.10. In Vitro Drug Release Studies

SLC release from prepared SNTs was determined using USP dissolution test apparatus II (paddle type), DT 700 LH device, Erweka, GmbH DT 700 (Heusenstamm, Germany). The dissolution medium used consisted of 500 mL of simulated saliva fluid without enzymes (SSF) of pH 6.8 at 37 °C ± 0.5 °C. The paddles stirring speed was 75 rpm and samples of 5 mL were withdrawn at various intervals of time, and replaced with fresh dissolution medium. Withdrawn samples were filtered through a 0.45-μm Millipore filter and analyzed for drug content and the experiments were carried out in triplicates.

### 2.11. In Vivo Pharmacokinetic Studies

A randomized, parallel, single-dose, open-label design with 14 days of screening and a 1-day study period was used. A single dose of each SLC-SNT equivalent to 50 mg (test) was given to selected human volunteers, who were instructed to keep the tablet in the oral cavity for 3 min before ingestion; the commercial SLC tablets (50 mg) were ingested orally with 250 mL of potable water. Volunteers remained at the site for approximately 36 h after sample administration so that blood samples could be collected as required [35]. All of these human studies were approved by the Ethics Committee of the Beni-Suef Clinical Laboratory Center for clinical studies (Approval No: 12-004-2020), Egypt. The studies were performed as per the Helsinki agreement protocol. The resulting data was analyzed by running a non-compartmental model in PK-SOLVER^®^ software to determine their different pharmacokinetic parameters.

## 3. Results and Discussion

### 3.1. Solubility Studies

**Figure 1:**
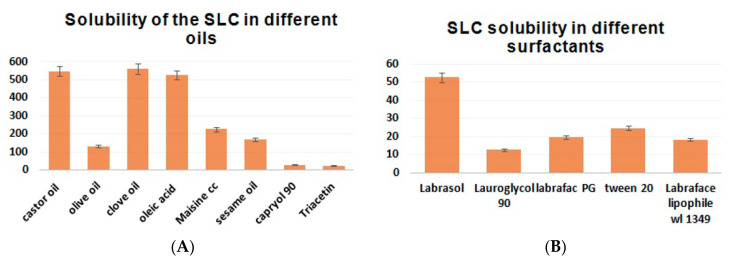

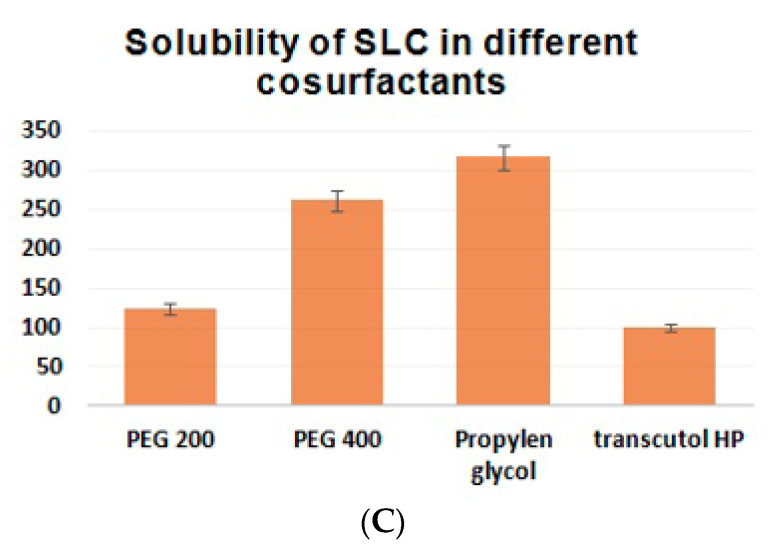
The solubility of SLC in various oils, surfactants, and cosurfactants.

SLC solubility in different oils, surfactants, and cosurfactants is depicted in Figure 1. A higher solubility of SLC was observed with clove oil (560.2 ± 28.01 mg/mL) and oleic acid (525.1 ± 26.25 mg/mL) (Figure 1A), with the surfactant Labrasol (52.56 ± 2.63 mg/mL) (Figure 1B), and with the cosurfactant propylene glycol (317.4 ± 15.87 mg/mL) (Figure 1C). The surfactants Tween20 and Labrafac PG, in addition to the cosurfactant PEG-400, also resulted in a significant rise in SLC solubility (Figure 1).

The solubility of the drug in the NE depended mainly on the ability of the oils to prevent drug “bleeding” or precipitation [36]. Different emulsion formulations were prepared for starting with oils, surfactants, and cosurfactants, which have a higher solubilized power of SLC and were mixed in different ratios to determine their homogeneity and dispersibility within the media. It was found that there was no relation between the solubilizing and dispersing abilities of each emulsion component. This was in agreement with a previously published paper [37]. Clove oil reduced the interfacial tension between the drug particles and facilitated the formulation and stabilization of small drug particles [38]. Oleic acid is a penetration enhancer, and it was found that the clove oil/oleic acid mixture (1:1) enhanced the solubility of SLC in the oil phase; this was the most significant criterium for prolonging stability and reducing the risk of drug particle precipitation. For surfactants, the second choice of surfactant (Tween 20) was used instead of Labrasol and mixed with the clove oil/oleic acid mixture and propylene glycol, which gave it the most dispersible, most homogenized, clearest emulsion. Tween 20 was used as a surfactant for its high hydrophile–lipophile balance values, which are due to its high level of adsorption on a droplet’s surface; this reduces its diameter rapidly [39]. The category and amount of surfactant used reflect on the performance of the self-nanoemulsifying system, because they improve the drug solubility and release of the lipophilic drug upon aqueous dilution [40]. Cosurfactants such as propylene glycol, which were used to enhance the emulsification of the surfactant [41], increased the entrapment of SLC particles and induced their solubility and stability [42]. Additionally, when 30% of the propylene glycol was present in the formulation, the smallest droplet and the high level of transparency were investigated [43]. In the present study, the mixture of clove oil/oleic acid, Tween 20, and propylene glycol was selected to formulate the SLC-SNEDDS.

### 3.2. Pseudoternary-Phase Diagram for SLC in Various Solvent Systems

The ternary-phase diagram for SLC was constructed and used to establish the mixture design to identify the levels of SNED components required to form the emulsion with a nanosized dispersed phase on dilution with water.

**Figure 2:**
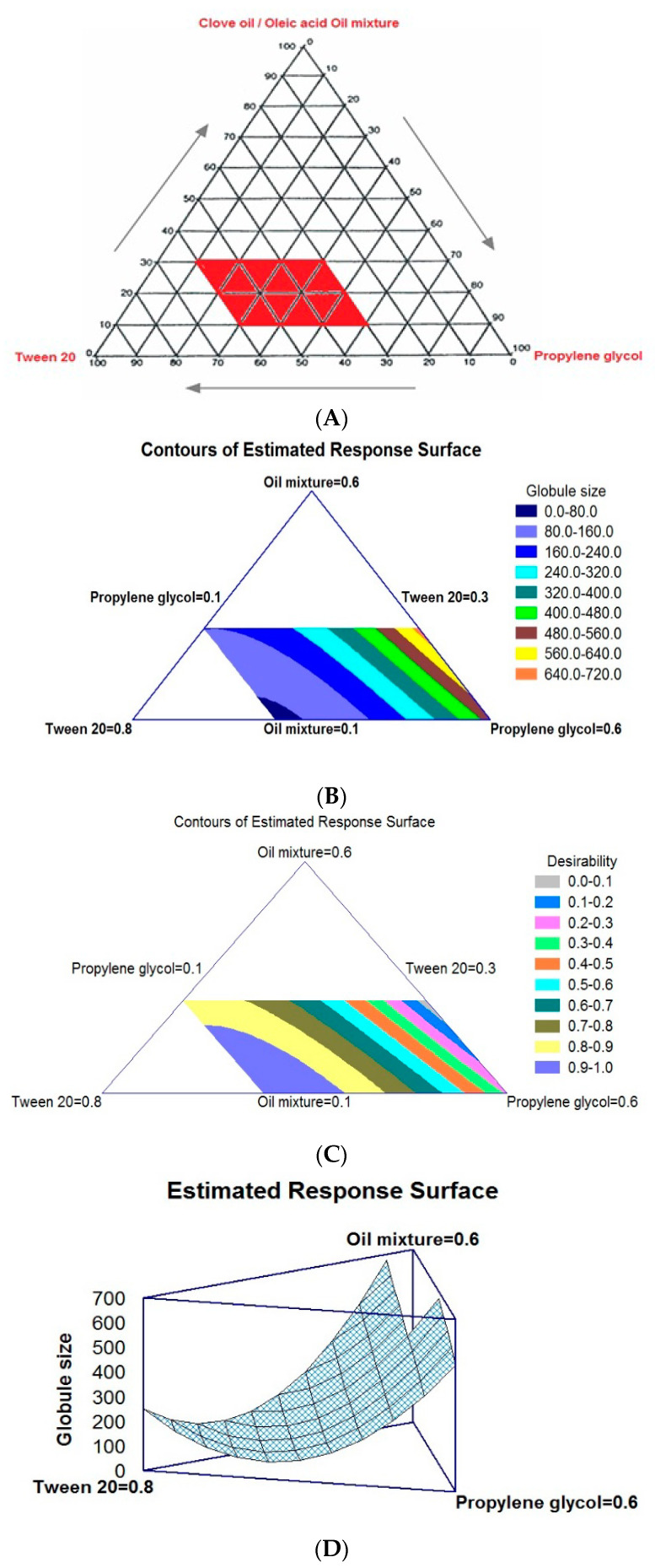
(**A**) Pseudoternary-phase diagram for SLC in various solvent systems (area in red is the NE region). (**B**,**C**) Two-dimensional contour plots and (**D**) response surface plot showing the effect of variables on selected responses of SLC-SNEDDS.

The different levels of the mixture components present in the area where the instant NE formation can be seen after dilution were found to be 10% to 30% (*w*/*w*) for oil, 30% to 60% (*w*/*w*) for surfactant, and 10% to 60% (*w*/*w*) for cosurfactant (Figure 2A).

### 3.3. Optimization of SLC-SNT Formulation

#### 3.3.1. Effect of Mixture Components on Globule Size

The projected experimental runs and their observed responses are listed in Table 2. The globule size of all the trial formulations was found to be in the range of 65.07 nm (NE-3) to 855.5 nm (NE-5), and these results were fitted to the cubic model. The ANOVA further confirmed the statistically significant relationship between the components and the response as evident from the 95% confidence interval and *p*-value of <0.05). The mixture component effect was further observed in the two-dimensional contour plots, as demonstrated in Figure 2B. The three corners and center of the triangle represent the positions of the three components and the mixture in equal parts. The few white regions are not fit to apply the regression analysis due to its component restraint. Accordingly, an alteration in the composition mixture can affect the change in globule size. The derived regression equation for an investigated response for the best-fit cubic model is given in Equation (2).

Globule size (Y_1_) = 863.006 X_1_ + 248.192 X_2_ + 514.249 X_3_ − 1384.67 X_1_ X_2_ + 76.6057 X_1_ X_3_ − 1185.61 X_2_ X_3_ − 871.765 X_1_ X_2_ X_3_ (2)

The two-dimensional contour plot and the regression equation confirmed that elevated concentrations of the surfactant and lower proportions of the oil and cosurfactant result in the formation of globules with the desired size. The minimum globule size that could be attained with the mixture design is represented in the black–blue area in the corner of the triangle (closer to the surfactant corner).

Our result is in agreement with the result reported in another research study, which found that increasing the oil phase percentage in the media may lead to globular enlargement as the specific surface area decreases and polydispersity of the emulsion increases [44]. The eugenol present in clove oil is responsible for the decrease of particle size to within the optimum nanosize [45]. The type and concentration of the surfactant are factors affecting the particle size. In the case of a higher surfactant concentration, the globular size decreases as the interparticle attraction decreases. Moreover, some types of surfactants played a critical role during the preparation of the NE (e.g., the glycerol surfactant produced an ultrafine globular size lower than 50 nm to form a self-nanoemulsifying system) [46]. Propylene glycol is considered as an organic solvent that helps in dissolving higher concentrations of surfactants and lipophilic drugs [47]. Our research is in agreement with those who found that there is an improvement in the stability of the SLC particle and its tolerance of dilution in the gastric media when it is prepared using a SNED.

#### 3.3.2. Response Optimization Using Desirability Approach

The effect of independent variables on the desirability function was demonstrated by contour plots, after optimizing the NE preparation to assume the mixture region (Figure 2D). Higher and lower proportions of X_1_ and X_1_. X_3_ components can maximize the desirability of a formulation, as demonstrated in the triangular contour plot. The violet area in the system represents the maximum desirability of the formulation. The optimized formula of the SLC-loaded NE with the minimum globule size was used in the preparation of SLC-SNTs. The formulation composition of NE-16 resulted in the formation of NE with the minimum globule size and, hence, was used for the preparation of SLC-SNTs (Table 2).

#### 3.3.3. Impact of NE Components on the Stability Index

Stability index data confirm the impact of the cosurfactant on the stability of the NE. The results were in the range of 59% (NE-3) to 91% (NE-2). The high stability of NE-3 can be credited to a high level of cosurfactant. Formulations with lower levels of cosurfactant suffered from stability issues. An extremely low interfacial tension can account for the thermodynamic stability of an NE; therefore, surfactants alone cannot decrease the interfacial tension to the desired level. The addition of a cosurfactant is essential in producing thermodynamically stable systems [48]. This result was further supported by Chen J et al., who worked on the application of cosurfactant to enhance the stability of anthocyanin microemulsion-based systems [49].

### 3.4. Morphologic Characterization

**Figure 3:**
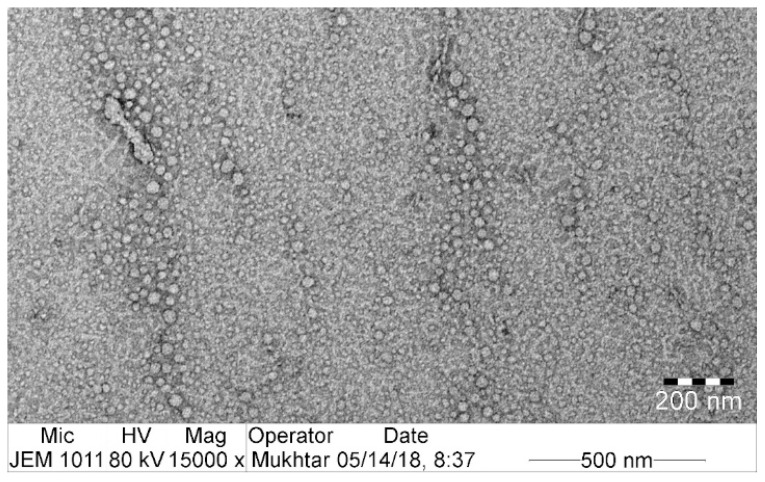
TEM image of optimized formulation SLC-SNEDDS.

The size of the globules is the critical factor in the development of SNEDDS, because the globule size can impact the drug release profile and absorption [50]. Colloidal dispersion showed a uniform globule size distribution (<100 nm), and no aggregation was observed, as portrayed in the TEM image (Figure 3). The measured globule size is in accordance with previous results (Table 2).

### 3.5. Formulation of SLC-SNTs

**Figure 4:**
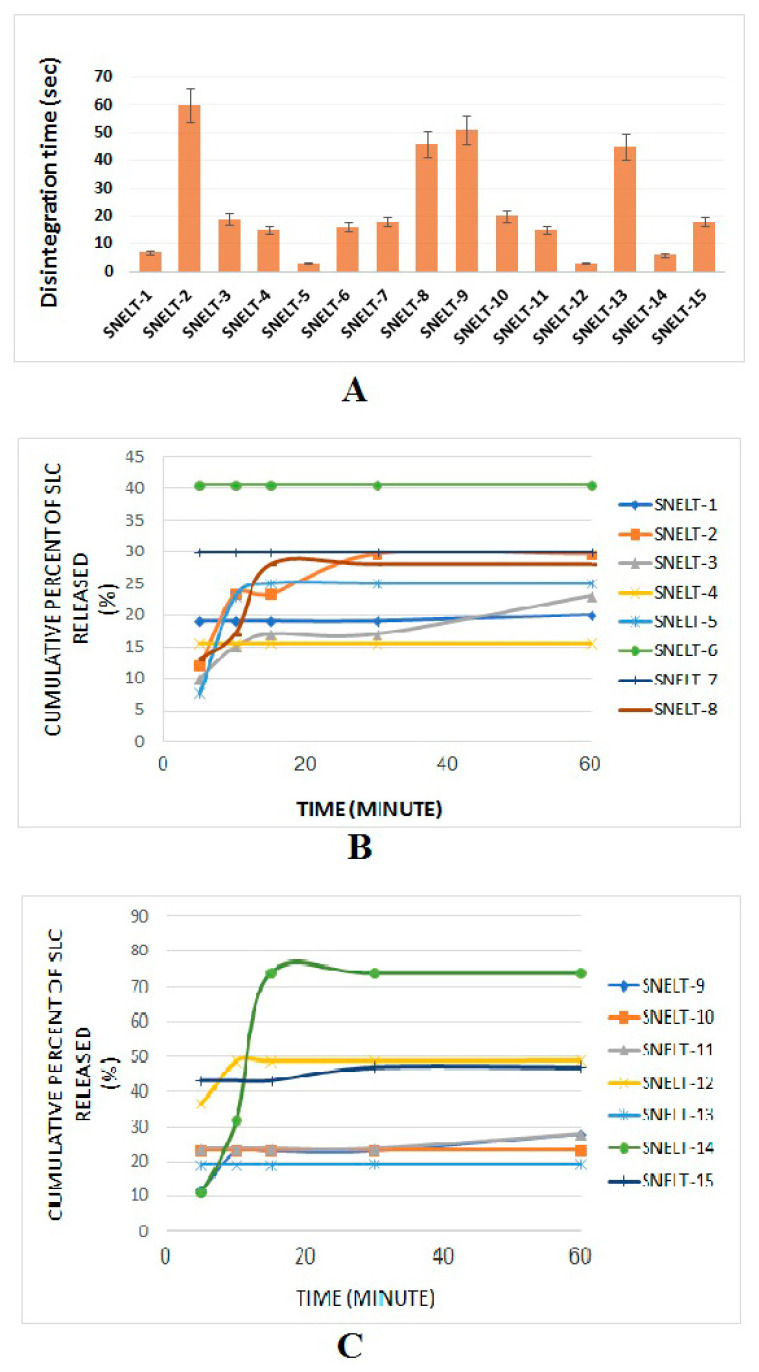
Disintegration and in vitro dissolution profiles of self-nanoemulsifying lyophilized tablets (SNTs).

SLC-SNTs were successfully prepared with a diluent called fumed silica. It acts as a binder to produce the best-quality tablets. The formula containing fumed silica demonstrated the best drug release profile and lowest disintegration time. Fifteen formulations (Table 3) were prepared as per experimental runs projected by the Box–Behnken design and evaluated for disintegration time (Figure 4A) and dissolution profile (Figure 4B,C). HPMC was added to prevent sedimentation of the solid nanoparticles via an increase in the viscosity of SNEDDS and gave required hardness to the SNTs. This could improve the stability of the SNTs [51]. Explotab, as a diluent, disintegrates, binds, and lubricates as reported in a previous study of preparation and evaluation of clopidogrel tablets [52].

Fumed silica, although insoluble, has a nanosized grade with a larger surface area that can enhance the amount of adsorbed drugs. This, in turn, can facilitate SLC distribution and absorption from the mucosal membrane of the buccal cavity [53].

Avicel^®^ PH 101 was added as a dissolution enhancer. It speeds the disintegration of tablets in two ways, wicking and swilling [52]. Mannitol is an excipient used mainly in fast-dissolving tablets and acts as a taste-masking sweet ingredient [54,55].

### 3.6. Evaluation of SLC-SNT Wetting Time

The wetting time study of SNT formulations showed times of 50 ± 34 to 180 ± 60 s, which indicated the effect of SNT contents and their concentrations on the tablet dissolution inside the oral cavity [56,57].

### 3.7. Disintegration Study

**Figure 5:**
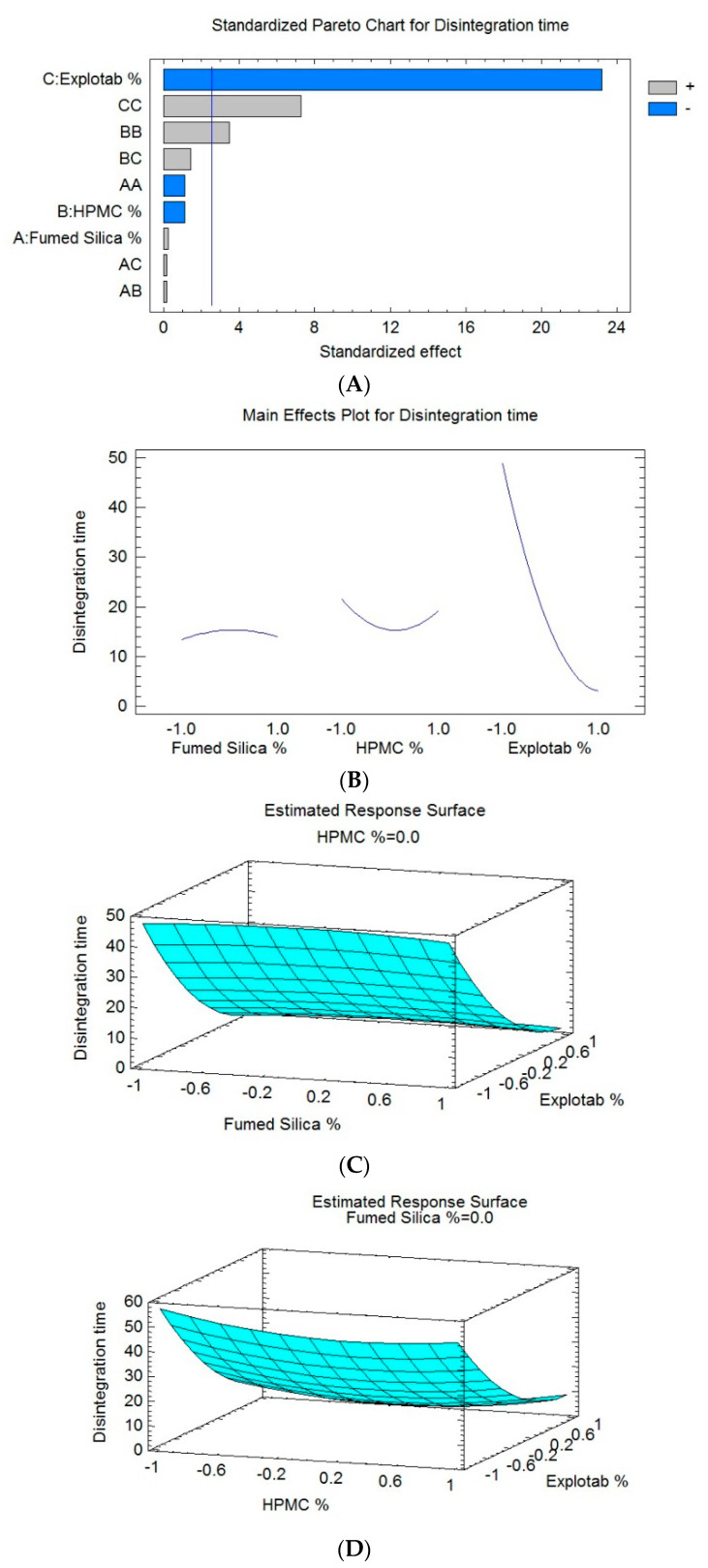
Pareto chart and response surface plots presenting the effect of mixture components on the disintegration time.

A broad range of disintegration times (from 3 s for SNT-5 to 60 s for SNT-2) was observed, as demonstrated in Table 4. An inverse and direct relationship was observed between the concentration of Explotab (X_3_) and silica (X_1_) for the disintegration time. The concentration of HPMC (X_2_) had a minor effect on (Y_1_) [58,59]. A shorter disintegration time was observed for SNT-5 and SNT-9 due to the presence of the lowest level of X_1_ and the highest level of X_3_ or X_2_. Accordingly, SNT-2 and SNT-9 were shown to have the longest disintegration time. All these results were in conformity with findings reported by Awal P et al. (Figure 5A).

### 3.8. In Vitro Dissolution Study

An in vitro drug release profile of SNT formulations is depicted in Figure 4B,C Regarding the curve of the cumulative release, it was found that the cumulative release of SLC from SNTs after 60 min was between 15.5% for SNT-4 and 74% for SNT-14. It was found that there were variabilities between different dosage forms, and this indicated that the Box–Behnken design showed the effect of factor variation on the values (Y_1_) and (Y_2_). This result might be attributed to a close relationship between the disintegration time and the amount of dissolution; this is reasonable, because the rate of release in drugs with the shortest disintegration time is faster than the drug excretion rate, and for this reason, the drug concentration in the blood becomes elevated over time [60].

### 3.9. Optimization of SLC-SNT Preparation by Response Surface Methodology

Optimization of SLC-SNTs was carried out by the Box–Behnken design, with the constraint of a low disintegration time and a maximum SLC drug release within 1 h. The projected experimental runs with different levels of components are compiled in Table 3. The quantitative effect of selected components was studied by applying the ANOVA. The obtained data were subjected to regression analysis to generate polynomial equations. In general, a *p*-value of less than 0.05 signified the model terms. Synergistic and antagonist effects can be identified by the positive and negative values of corresponding terms, respectively. The special cubic model showed a good correlation with the experimental results, which was evident from high R^2^ and adjusted R^2^ values (99.1828-Y_1_ and 99.5453-Y_2_, respectively). A high correlation between the experimental and predicted data was obtained when the results were expressed graphically with the given data.

#### 3.9.1. Effect of Formulation Variables on Disintegration of SNTs

As required, fast-disintegration tablets should have an immediate breakdown into small-sized granules/fragments in order to yield the maximum surface area that can be offered for dissolution media [61]. SNT formulations showed significant variations in their disintegration time. The effect of independent factors on the disintegration time was further demonstrated by three-dimensional response surface graphs (Figure 5B–D). The generated polynomial equation is given in Equation (3).

In vitro disintegration time (Y_1_, s) = 15.3333 + 0.25 X_1_ − 1.125 X_2_ − 22.875 X_3_ − 1.66667 X_1_^2^ + 0.25 X_1_X_2_ + 0.25 X_1_X_3_ + 5.08333 X_2_^2^ + 2.0 X_2_X_3_ + 10.5833 X_3_^2^ (3)

The ANOVA (Table 4) and Pareto chart (Figure 5A) depict a considerable negative impact of X_3_ on the disintegration time of the SLC-SNTs (Y_1_), with a *p*-value of 0.0000. This result confirmed the antagonistic relationship between this factor and the disintegration time. An increase in the concentration of the Explotab made the tablet disintegrate rapidly. However, the ANOVA confirmed the major effect of the interaction term (X_2_X_2_) (X_3_X_3_) corresponding to the disintegration time of the SLC-SNTs (Y_1_), with *p*-values of 0.0173 and 0.0008, respectively. As a result, the higher proportions of Explotab (X_3_) minimized the disintegration time (Y_1_) of the formulations. Both the fumed silica (X_1_) and the HPMC (X_2_) had a minor effect on the disintegration time (Y_1_). The percentage of Explotab mainly affected the disintegration time, whereas the HPMC and fumed silica had minor effects. By comparing the values in Table 4, it can be found that when the percentage of the Explotab (X_3_) changed while the percentage of fumed silica (X_1_) and HPMC (X_2_) were fixed, the disintegration time (Y_1_) was much increased, from 7 s in SNT-1 to 60 s in SNT-2. When the value of (X_2_) changed and the values of (X_1_) and (X_3_) were fixed, as in SNT-1 and SNT-14, the disintegration time (Y_1_) was not significantly affected by (X_2_). The same previous result was obtained when the value of (X_1_) changed while the values of (X_2_) and (X_3_) were fixed in both SNT-5 and SNT-12.

As a result, formulations consisting of a low level of either X_1_ or X_2_ and a high level of X_3_ showed shorter disintegration times. Enhanced disintegration can be credited to the ample concentration of Explotab, as it favors the high porosity of tablets and makes the tablet swell and break down as quickly as possible. Because of the hydrophilic nature of the Explotab, it can absorb many times its weight in the presence of a liquid medium. Subsequent to this, the tablet–air interface will be spontaneously replaced by the tablet–water interface and will retain capillary flow, leading to rapid disintegration. Conversely, SNT formulations with lower levels of X_1_ and higher levels of X_2_ or X_3_ showed a longer disintegration time. Other related factors were used as structure-forming agents. Additionally, the bonding structure and pore structure affect the disintegration time. The formed pores can facilitate the quicker penetration of water and burst the tablets into smaller fragments. Explotab acts as a binder on a high concentration by forming a viscous barrier, which accelerates the tablet disintegration time [55]. A superdisintegrant such as the Explotab enhances the bioavailability of the tablets by facilitating the drug release; it increases the porosity and fluid penetration of the tablets via capillary action [62]. The binding effect of HPMC decreases the porosity and diminishes the process of disintegration. This may be due to a high level of cross-linking.

#### 3.9.2. Effect of Independent Variables on the Percentage of Release of SLC after 5 min (Y_2_)

Dissolution profiles of prepared SLC-SNT formulations are shown in Figure 4. SLC release after 5 min from the formulations demonstrated a significant variation in the range of 16% (SNT-15) to 68% (SNT-12). The generated polynomial equation for this response is given below:

% release after 5 min (Y_2_, %) = 15.0 + 21.375 X_1_ + 0.5 X_2_ + 3.875 X_3_ + 13.75 X_1_^2^ + 0.75 X_1_X_2_ + 3.5 X_1_X_3_ − 1.0 X_2_^2^ + 0.25 X_2_X_3_ + 1.25 X_3_^2^ (4)

**Figure 6:**
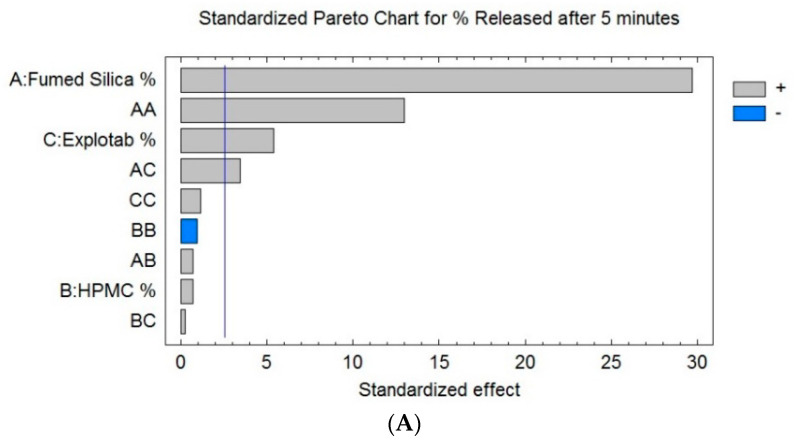

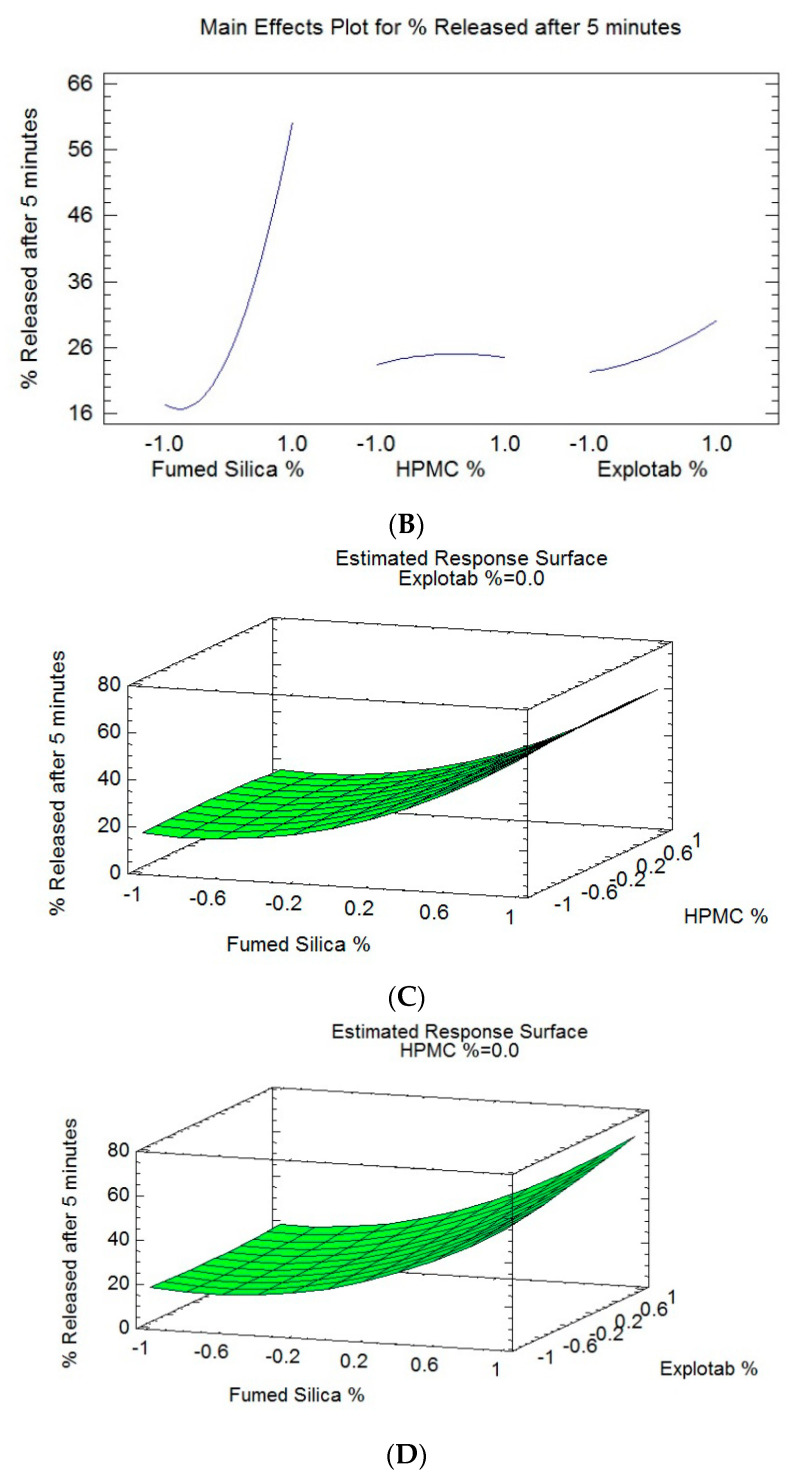
Pareto and response surface graphs showing the connection between selected variables and SLC release.

Statistical analysis (Table 4) and the Pareto chart (Figure 6A) show a significant positive effect of X_1,_ X_3,_ and the interaction terms (X_1_X_1_) and (X_1_X_3_) on the disintegration time of SLC-SNTs (Y_1_), with *p*-values of 0.0000, 0.0030, 0.0000, and 0.0185, respectively. This reveals the direct relation between X_1_, X_3,_ and Y_2_, i.e., when the percentage of both fumed silica and the Explotab increase, the percentage of release after 5 min (Y_2_) will increase.

The effect of selected components on the cumulative drug release was confirmed by the three-dimensional response surface plots (Figure 6B–D). It was evident that the dissolution profile of SLC-SNTs containing the highest proportions of fumed silica (X_1_) and Explotab (X_3_) showed the highest percentage of release after 5 min (Y_2_). The HPMC (X_2_) had no significant effect on (Y_2_). By comparing the values in Table 4 to evaluate the effect of the factors on the value, it was found that when the percentage of fumed silica (X_1_) changed while the percentage of HPMC (X_2_) and the Explotab (X_3_) was fixed, the percentage of release after 5 min (Y_2_) increased from 18% in SNT-5 to 68% in SNT-12. This indicated that (X_1_) significantly affected the percentage of release after 5 min (Y_2_). Additionally, it was seen that when the value of (X_3_) changed and the values of (X_1_) and (X_2_) were fixed, the value of the percentage of release after 5 min (Y_2_) varied between 20% in SNT-2 and 29% in SNT-1, and this indicated that (X_3_) had a small positive effect on (Y_2_). When the value of (X_2_) changed and the values of (X_1_) and (X_3_) were fixed, the percentage of release after 5 min (Y_2_) changed from 29% in SNT-1 to 31% in SNT-14; this indicated that (X_2_) did not affect (Y_2_).

The dissolution profile of SLC-SNT formulations containing maximum and minimum concentrations of both factors X_1_ and X_3_, respectively, accounted for the highest and lowest cumulative release of SLC, respectively. Fumed silica acted as an inert carrier and adsorbed the lipophilic drugs, which improved the drug release [35]. Fumed silica-based drug delivery systems have shown considerable promise for improving the oral delivery of poorly water-soluble drugs owing to the high surface areas with associated ability to physically adsorb high-drug loads in a molecular or amorphous form. This in turn allows the molecular state drug release in aqueous gastrointestinal environments, potential for supersaturation, and hence facilitates enhanced absorption and increased bioavailability. It also acted as a diluent and good binder to produce the best-quality tablets in terms of the dissolution rate [50]. Formulations containing high or low levels of X_3_ and X_1_ resulted in the lowest cumulative release of SLC. Because the drug dissolution directly depends on tablet disintegration, higher superdisintegration concentrations favor a higher cumulative release of SLC. SNTs will break into smaller particles and more surface area will be exposed to the dissolution medium, finally triggering a maximum cumulative drug release of SLC.

#### 3.9.3. Optimization

Concentrations of independent variables are optimized by fixing the targets for every response and then creating the overlay graph. The desirability function (D) was concurrently applied to optimize the models obtained from the statistical analysis [63,64]. Selected variables were integrated into the design space, and for simultaneous optimization, each response had a value assigned to each goal. The disintegration time was set to minimum, and the SLC release was adjusted to maximum (at the end of 1 h). Thus, D was projected for the responses using a nondimensional scale. Plots of desirability show assorted colors expressing the assortment of D values, in the range of 0 and 1. To optimize the study statistical analysis, a global D was applied. On the basis of these criteria, 0.4% of fumed silica, 0.1% of HPMC, and 0.4% of the Explotab were found to be the optimized levels that could accomplish the prerequisites of the formulation. The optimized formulation had the maximum desirability function-1. The optimized formulation exhibited a 30-s disintegration time, and the SLC drug release was found to be 68% within 5 min. All these experimental results were in reasonable agreement with the predicted values, and the relative error was found to be less than 5%. This affirms the accuracy of the applied design. All the results can favor enhancing oral bioavailability [65].

### 3.10. Bioavailability Studies in Healthy Human Volunteers

**Figure 7:**
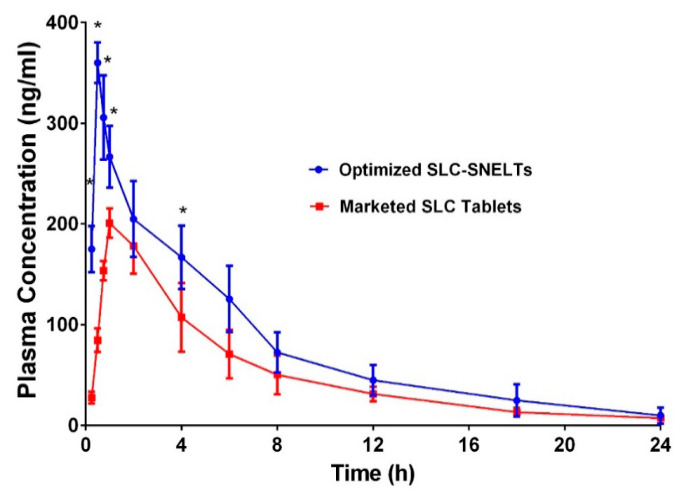
Comparative plasma concentration–time profiles of optimized formulation and marketed formulation (mean ± SD, *n* = 3).

The plasma concentration–time profile of the optimized SLC-SNTs through oral administration was compared with that of the marketed SLC formulation to confirm the enhancement of the pharmacokinetic profile (Figure 7). All the selected volunteers completed the study. During the study, no adverse reactions were reported. Collected samples were analyses as per the method protrayed by Reham Zayed et al. [66]. The complete pharmacokinetic parameters are summarized in Table 4. The results confirm the shorter T_max_ (30 min) and enhanced maximum plasma concentration (C_max_ 360 ± 20 ng/mL) of the SLC-SNT formulation in contrast to the marketed formulation (T_max_ 30 min; C_max_ 205 ± 11 ng/mL). All these observations confirm the improved rate and absorption of SLC (Table 5). Furthermore, the optimized formulation enhanced the AUC. The relative bioavailability of the test formulation was 144.28% compared with the marketed formulation. This was in accordance with the work by Hosny KM et al., where enhancement in the bioavailability was observed by >1.87 fold [67]. All these obtained results clearly suggest that the incorporation of SLC in the SNT system can enhance the oral bioavailability of SLC. The enhanced SLC absorption was probably due to increased solubilization via a decrease in the globular size, which subsequently maximized the surface area. In general, drug absorption is considered a rate-limiting step. The dissolved SLC was directly absorbed from the oral cavity, thus bypassing the rate-limiting step. Furthermore, an enhanced dissolution rate can lead to a noteworthy increase in absorption and improved oral bioavailability [68].

## 4. Conclusions

The current study establishes the significance of SNTs in improving the oral bioavailability of SLC for effective clinical usage. The optimized SLC-SNT is composed of a 0.4%/10% clove oil/oleic acid mixture, 60% Tween 20, and 30% propylene glycol with a 65.07-nm globule size. The Box–Behnken design was effectively employed for the optimization of independent factors to produce SNTs with a quicker disintegration time and the highest drug release. The optimized SLC-SNT formulation was composed of 0.4% of fumed silica (X_1_), 0.1% of HPMC (X_2_), and 0.4% of Explotab (X_3_) with the addition of 0.1% of Avicel^®^ PH 101 and 0.1% of mannitol. The optimized formulation had a disintegration time of just 3 s and a release time of 68% of the SLC by the end of 1 h. Relative bioavailability and pharmacokinetic parameters of the optimized formulation in healthy volunteers showed a remarkable improvement in the bioavailability of SLC compared with the marketed formulation. All these results suggest that SLC-SNTs can be used in the management of ED owing to their ability to enhance bioavailability.

## Figures and Tables

**Table 1:** Experimental plan of mixture design (component levels and selected response).

Component	Level		Response
	Low	High	
Oil percentage; (X_1_)	0.1	0.3	Mean globule size
Surfactant percentage; (X_2_)	0.3	0.6	
Co-surfactant percentage; (X_3_)	0.1	0.6	

**Table 2:** Projected trial formulation compositions and the observed response of sildenafil citrate (SLC)-loaded nanoemulsion (NE) formulations, as recommended by the mixture design.

Formulation Code	Independent Variables (Mixture Components)			Dependent Variables (Responses)
	X_1_ (gm)	X_2_ (gm)	X_3_ (gm)	Y_1_ (nm)
NE-1	0.1	0.3	0.6	528.17
NE-2	0.3	0.3	0.4	233.6
NE-3	0.1	0.6	0.3	65.07
NE-4	0.3	0.6	0.1	382
NE-5	0.15	0.375	0.475	855.5
NE-6	0.25	0.375	0.375	199.5
NE-7	0.15	0.525	0.325	165.84
NE-8	0.25	0.525	0.225	470
NE-9	0.2	0.3	0.5	720.49
NE-10	0.1	0.45	0.45	135.09
NE-11	0.3	0.45	0.25	296.22
NE-12	0.2	0.6	0.2	602.21
NE-13	0.2	0.45	0.35	532.56
NE-14	0.1	0.3	0.6	300.5
NE-15	0.3	0.3	0.4	512.4
NE-16	0.1	0.6	0.3	103.5

**Table 3:** Concentrations of independent factors (percentage) in a randomized order for formulating SLC-SNTs.

Formula Code	X_1_	X_2_	X_3_
SNT-1	2	0	4
SNT-2	2	0	0
SNT-3	4	0	2
SNT-4	2	1	2
SNT-5	0	1	4
SNT-6	2	1	2
SNT-7	0	0	2
SNT-8	0	1	0
SNT-9	2	2	0
SNT-10	4	2	2
SNT-11	2	1	2
SNT-12	4	1	4
SNT-13	4	1	0
SNT-14	2	2	4
SNT-15	0	2	2

Abbreviations: X_1_, fumed silica percentage; X_2_, HPMC percentage; X_3_, Explotab percentage.

**Table 4:** ANOVA of the responses Y_1_ and Y_2_.

Factors	Disintegration Time (Y_1_), Second			Cumulative Release after 5 min (Y_2_), %		
	Estimate	F-Ratio	*p*-Value	Estimate	F-Ratio	*p*-Value
X_1_	0.25	0.06	0.8100	42.75	880.75	0.0000 *
X_2_	−1.125	1.30	0.3057	1.0	0.48	0.5185
X_3_	−22.875	537.83	0.0000 *	7.75	28.95	0.0030 *
X_1_X_1_	−1.66667	1.32	0.3029	27.5	168.21	0.0000 *
X_1_X_2_	0.25	0.03	0.8648	1.5	0.54	0.4946
X_1_X_3_	0.25	0.03	0.8648	7.0	11.81	0.0185 *
X_2_X_2_	5.08333	12.26	0.0173 *	−2.0	0.89	0.3889
X_2_X_3_	2.0	2.06	0.2111	0.5	0.06	0.8159
X_3_X_3_	10.5833	53.13	0.0008 *	2.5	1.39	0.2914
R^2^	99.1828			99.5453		
Adj. R^2^	97.7119			98.7269		
SE	2.78986			2.03715		
MAE	1.32222			0.933333		

Note: * Significant factors; Abbreviations: X_1_, fumed silica percentage; X_2_, HPMC percentage; X_3_, Explotab percentage; SEE, standard error of estimate; and MAE, mean absolute error.

**Table 5:** Comparative pharmacokinetic parameters of optimized formulation and marketed tablets (mean ± SD, *n* = 6).

PK Parameters	Optimized SLC-SNTs	Marketed SLC-Tablets
C_max_ (ng/mL)	360 ± 20	205 ± 11
T_max_ (min)	30 ± 0.0	90 ± 6.0
t_1/2_ (h)	4.46 ± 1.46	6.2 ± 1.1
AUC _0-t_ (ng/mL h)	1801 ± 400	1248.25 ± 233.125
AUC_0-inf_ (ng/mL h)	1846.36 ± 727.34	1281.5 ± 316.82
AUMC _0-inf_ (ng/mL h^2^)	11,801.119 ±10,058.3	7185.7 ± 1398.60
K_el_ (h^−1^)	0.154 ± 0.02	0.110 ± 0.040
MRT (h)	6.39 ± 2.10	7.16 ± 1.3
Relative BA (%)	144.28%	-
